# Daily intake of heat-killed *Lactobacillus plantarum* L-137 improves inflammation and lipid metabolism in overweight healthy adults: a randomized-controlled trial

**DOI:** 10.1007/s00394-019-02112-3

**Published:** 2019-10-16

**Authors:** Yusuke Tanaka, Yoshitaka Hirose, Yoshihiro Yamamoto, Yasunobu Yoshikai, Shinji Murosaki

**Affiliations:** 1 Research Division, Research and Development Institute, House Wellness Foods Corp., Imoji 3-20, Itami, Hyogo 664-0011 Japan; 2 Division of Host Defense, Medical Institute of Bioregulation, Kyushu University, Fukuoka, 812-8582 Japan

**Keywords:** *Lactobacillus plantarum*, Heat-killed bacteria, Inflammation, Lipid metabolism, Overweight subjects

## Abstract

**Purpose:**

The effects of heat-killed *Lactobacillus plantarum* L-137 (HK L-137) on inflammation and lipid metabolism were investigated in overweight volunteers.

**Methods:**

One hundred healthy subjects with a body mass index from 23.0 to 29.9 (51 men and 49 women; mean age: 41.4 years) were enrolled in this randomized, double-blind, placebo-controlled, parallel group study. Subjects were randomly assigned to daily administration of a tablet containing HK L-137 (10 mg) or a placebo tablet for 12 weeks. Blood samples were collected every 4 weeks to measure biomarkers of lipid metabolism and inflammatory mediators.

**Results:**

The percent change of concanavalin A-induced proliferation of peripheral blood mononuclear cells was significantly larger in the HK L-137 group than in the control group, similar to previous studies. The decreases of aspartate aminotransferase and alanine aminotransferase over time were significantly larger in the HK L-137 group than in the control group, as were the decreases of total cholesterol, low-density lipoprotein cholesterol, and the leukocyte count at one time point. These effects of HK L-137 were stronger in the subjects with higher C-reactive protein levels.

**Conclusions:**

These findings suggest that daily intake of HK L-137 can improve inflammation and lipid metabolism in subjects at risk of inflammation.

## Introduction

Obesity is one of the most important public health issues in many countries. It has been suggested that obesity-related chronic inflammation contributes to various metabolic disorders, including insulin resistance [1], type 2 diabetes [2], non-alcoholic fatty liver disease [3], cardiovascular disease [4–7], hypertension [8], and dyslipidemia [9]. Production of inflammatory cytokines by adipose tissue is increased in the obese state, followed by elevation of the serum levels of inflammatory mediators, including C-reactive protein (CRP), plasminogen activator inhibitor-1, and the white blood cell count. There is considerable evidence that inflammatory cytokines like tumor necrosis factor (TNF)-α, interleukin (IL)-1β, and IL-6 not only induce systemic insulin resistance, but also influence lipid metabolism. For example, it was reported that these cytokines induce lipolysis in adipocytes [10, 11], as well as enhancing lipogenesis and triglyceride (TG) synthesis in hepatocytes, resulting in increased hepatic secretion of very-low-density lipoprotein (VLDL) and elevation of the serum TG level [12–15]. Therefore, suppression of inflammatory mediators in obese or overweight persons may be effective for prevention/improvement of obesity-associated metabolic disorders such as insulin resistance and dyslipidemia.

Several lactic acid bacteria are used as probiotics with health-promoting effects that include modulation of the intestinal flora, protection against intestinal infection, and immune modulation. Recent studies have indicated that probiotics can also modulate host lipid metabolism. Administration of viable *Lactobacillus plantarum* (*L. plantarum*) LIP-1 was reported to normalize lipid metabolism by modifying the gut microbiota in hyperlipidemic rats with dietary obesity [16]. Kefir is a fermented milk beverage containing viable bacteria, viable yeasts, and their products, which exhibits a cholesterol-lowering effect [17–21]. A meta-analysis of 11 clinical trials evaluating the effects of probiotics on serum lipids revealed that administration of probiotics decreased serum total cholesterol (TC) and LDL cholesterol (LDL-C), but had no effect on high-density lipoprotein cholesterol (HDL-C) or TG [22]. The mechanisms by which viable probiotic bacteria suppress dyslipidemia remain to be fully elucidated. Similarly, the reduction of serum cholesterol or TG by intake of some heat-killed bacteria has also been studied. Toshimitsu et al. reported that administration of heat-killed *L. plantarum* OLL2712 to KKAy mice decreased serum TG levels by suppressing chronic inflammation [23]. In hamsters on a high-fat diet, heat-killed *L. reuteri* GMNL-263 lowered the serum levels of TC, LDL-C, and TG, as well as the plasma malondialdehyde level, without decreasing HDL-C [24]. These results suggest that not only viable bacteria, but also heat-killed bacteria, can be used to improve obesity-induced inflammation and abnormalities of lipid metabolism.

*Lactobacillus plantarum* L-137 is a strain isolated from a fermented fish and rice dish that is popular in Southwest Asia [25]. It was reported that daily intake of heat-killed *L. plantarum* L-137 (HK L-137) improved health-related quality of life [26] and reduced the occurrence of upper respiratory tract infections [27] by its immunomodulatory effects. Recently, Uchinaka et al. demonstrated an anti-inflammatory effect of HK L-137 on the heart and adipose tissue in DahlS.Z-*Lepr*^fa^/*Lepr*^fa^ (DS/obese) rats, a model of metabolic syndrome. Administration of HK L-137 to these rats attenuated left-ventricular inflammation and fibrosis, reduced adipose tissue hypertrophy and inflammation, and improved insulin resistance [28]. These results suggested that HK L-137 might also ameliorate obesity-induced chronic inflammation and metabolic disorders in obese or overweight healthy persons.

Accordingly, we investigated the effect of daily intake of HK L-137 on immune function and serum levels of inflammatory and lipid markers in overweight healthy volunteers.

## Experimental methods

### Subjects

**Fig. 1:**
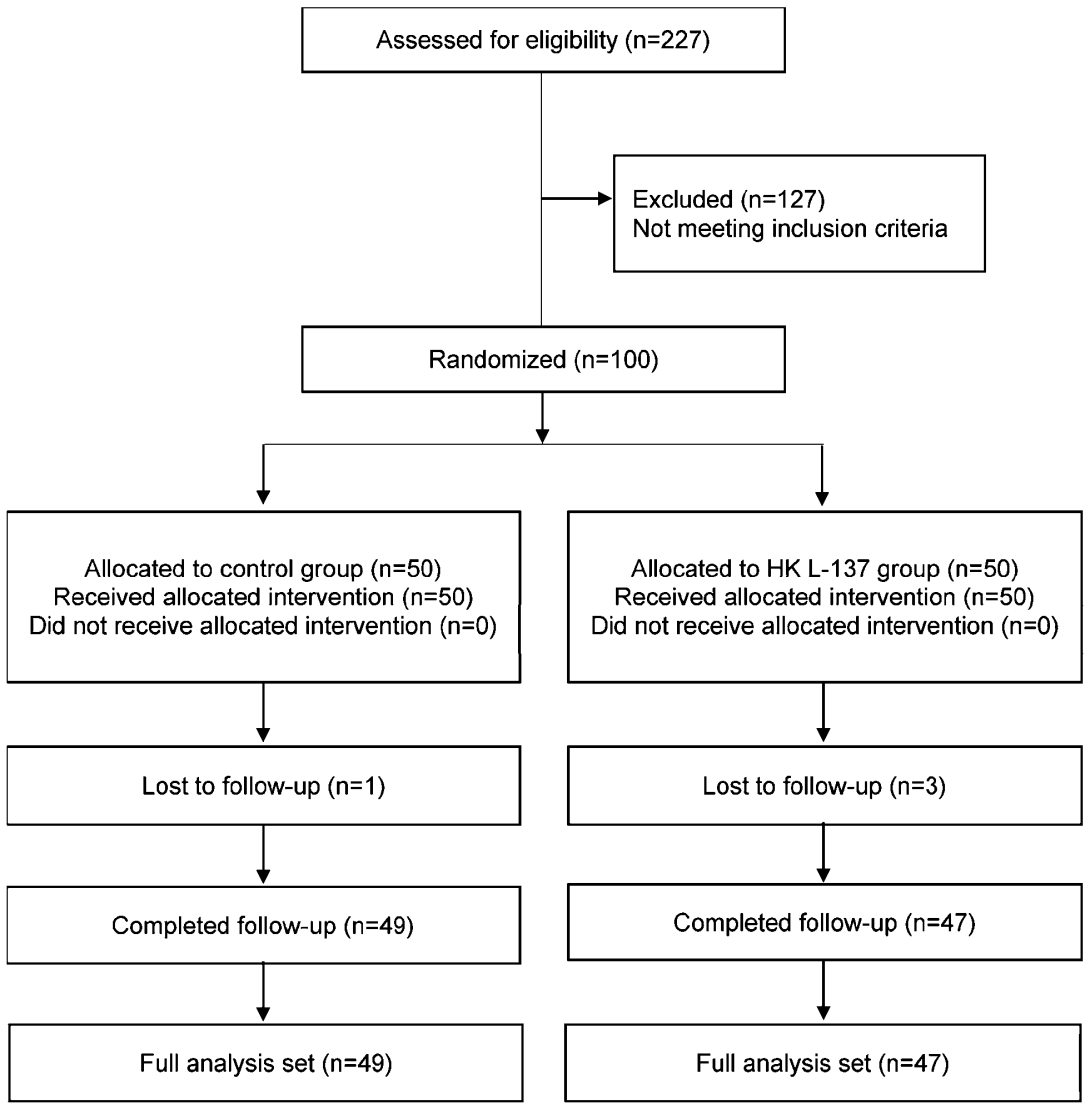
This flow diagram summarized according to the consolidated standards of reporting trials shows the number of subjects randomized, lost to follow-up, and analyzed by treatment arm

Healthy persons aged from 20 to 75 years whose body mass index (BMI) was more than 23 and less 30 were recruited in November 2017, and their eligibility for this study was assessed. Exclusion criteria included the following: allergic rhinitis or bronchial asthma; use of medications that could affect the results of this study; prior daily consumption of the lactic acid bacteria used in this study; pregnancy, breastfeeding, or intention to become pregnant; current or previous history of diabetes, hepatic disease, kidney disease, cardiac disease, gastrointestinal disease, vascular disease, or other diseases; high alcohol intake; heavy smoking; cow’s milk allergy; extremely irregular diet; unstable work schedule or working the night shift; and being judged unsuitable for this trial by the investigator or subinvestigator. Among 227 potential recruits, 100 persons (51 men and 49 women; mean age: 41.4 years) were found to be eligible and were randomly assigned to the HK L-137 group or the control group (Fig. 1). The sample size was determined from the results of a previous study investigating the effects of probiotics on serum total cholesterol [29]. In that study, the mean within-group changes showed a normal distribution with a standard deviation of 0.70. If the true difference of the mean value between the treated and control groups is assumed to be 0.45, we needed 39 subjects per group to be able to reject the null hypothesis (i.e., the population means of both groups are equal) with a power of 0.8. The probability of a Type I error associated with this test of this null hypothesis was 0.05. We initially recruited 100 volunteers to allow for an estimated drop-out rate of 20% over the study period. Eating habits were not considered in detail, but the subjects were instructed to remain on their habitual diet during the study. This study was registered with the University Hospital Medical Information Network Clinical Trials Registry (UMIN000030079).

### Preparation of HK L-137

LP20 (House Wellness Foods Corporation, Hyogo, Japan), containing 20% HK L-137 and 80% dextrin, was used. Preparation of HK L-137 for addition to LP20 was done as described previously [30].

### Experimental design

The 100 subjects were enrolled in a study with a randomized, double-blind, placebo-controlled, parallel group design. The subjects were randomly assigned to either group with stratification by age, sex, BMI, and CRP using sequentially numbered sealed envelopes that each contained one treatment randomly generated by a computer program. After grouping, the subjects either took one tablet per day containing 50 mg of LP20 or a matching control tablet that contained dextrin instead of LP20 for 12 weeks. Blood and urine samples were collected every 4 weeks to measure biomarkers of immune function and other parameters (lipids, glucose, and inflammatory mediators). Biomarkers of immune function (Con A-induced proliferation and the Th1:Th2 ratio) were the primary outcome variables and were measured by an external clinical laboratory (SRL, Tokyo, Japan). Biochemical tests, hematology tests, and urinalysis were done by another external clinical laboratory (LSI Medience, Tokyo, Japan). Inflammatory biomarkers (CRP, leukocyte count, and TNF-α) and lipid metabolism parameters (glucose, TC, LDL-C, and HDL-C) were assessed as secondary outcomes. This study was conducted at Higashi Koganei Sakura Clinic (Tokyo, Japan) from December 2017 to March 2018.

### Biomarkers of immune function

Peripheral blood mononuclear cells (PBMCs) were isolated by the Ficoll–Conray centrifugation technique (Ficoll–Conray, *d* = 1.077). Then, 5 × 10^5^ cells/mL were cultured for 72 h at 37 °C with or without the optimal dose of concanavalin A (Con A, 5 µg/mL), with pulse labeling by 37 kBq of [3H]-thymidine during the last 8 h of incubation. DNA synthesis was assessed by measuring thymidine uptake. The percentage of cluster of differentiation 4 (CD4) + T cells producing interferon (IFN)-γ and IL-4 (Th1:Th2 ratio) was determined by single-cell measurement of intracellular cytokines using flow cytometry, as described previously [31]. Serum TNF-α concentration was determined by LSI Medience using a Human TNF-α QuantiGlo ELISA Kit (R&D Systems, Inc.) according to the manufacturer’s instructions.

### Stratified analysis

Participants were stratified according to the serum CRP level, with the cut-off value being 0.045 mg/dL. Subjects with a serum CRP level < 0.045 mg/dL were assigned to the low CRP group and those with a serum CRP level ≥ 0.045 mg/dL were assigned to the high CRP group.

### Statistical analysis

IBM SPSS statistics version 25 software was used for statistical analyses. The percent changes of immune function parameters relative to baseline were analyzed by two-way analysis of variance (ANOVA), and the changes from baseline of other parameters were analyzed by two-way repeated ANOVA. For comparison between groups at each time point, the f test was performed to assess equality of variance, followed by the unpaired Student’s *t* test or Welch’s *t* test.

## Results

### Baseline characteristics

One subject in the control group and three subjects in the HK L-137 group dropped out for personal reasons, but the other 96 subjects completed the study and were included in the statistical analysis (Fig. 1). The baseline parameters of immune function, lipid metabolism, and inflammation of these 96 participants are shown in Table 1. Baseline characteristics did not differ between the two groups.

**Table 1:** Baseline characteristics of the subjects

	Control group	HK L-137 group
*N*	49	47
Sex^a^		
Male	26	23
Female	23	24
Age (years)^b^	42.1 ± 10.9	41.3 ± 10.2
BMI	26.1 ± 2.0	26.1 ± 1.6
Immune function parameters^b^		
Con A-induced proliferation (stimulation index)^c^	261 ± 108	220 ± 112
Th1:Th2 ratio	14.8 ± 9.3	15.3 ± 13.6
Blood parameters^b^		
AST (units/L)	20.6 ± 4.3	21.1 ± 7.5
ALT (units/L)	20.5 ± 8.2	21.9 ± 13.8
CRP (mg/dL)^d^	0.090 ± 0.128	0.106 ± 0.180
Glucose (mg/dL)	87.8 ± 7.9	85.4 ± 7.2
TC (mg/dL)	204 ± 29	207 ± 29
HDL-C (mg/dL)	54.4 ± 11.3	57.3 ± 15.9
LDL-C (mg/dL)	125 ± 27	123 ± 27
Leukocyte count (/mL)	5.59 ± 1.27	6.24 ± 2.11
TNF-α (pg/mL)	1.96 ± 0.87	2.03 ± 0.93
TG (mg/dL)	109 ± 61	124 ± 90

Values are the mean ± SD

*BMI* body mass index, *Con A* concanavalin A, *Th1* type 1 T helper, *Th2* type 2 helper, *AST* aspartate transaminase, *ALT* alanine transaminase, *CRP* C-reactive protein, *TC* total cholesterol, *HDL-C* high-density lipoprotein cholesterol, *LDL-C* low-density lipoprotein cholesterol, *TNF* tumor necrosis factor, *TG* triglyceride

^a^Comparison between two groups by the Chi-square test

^b^Comparison between two groups by the unpaired Student’s *t* test or Welch’s *t* test

^c^Ratio of Con A-induced proliferation to unstimulated proliferation

^d^Control group: *n* = 49; HK L-137 group: *n* = 45

### Effects of HK L-137 on immune function

In the control group, three sets of proliferation data (1 at 4 weeks and 2 at 8 weeks) were excluded from analysis because Con A-stimulation of PBMC elicited no response. The percent change of proliferation induced by Con A (stimulation index = Con A-stimulated cells/unstimulated cells) showed considerable variation, especially over time due to the complicated method of measurement [26]. However, it was significantly larger in the HK L-137 group than in the control group over time (*P* = 0.04) (Table 2), similar to the results of previous studies [26, 27].

**Table 2:** Changes of immune function relative to baseline

		Change vs. baseline (%)^a^			Two-way ANOVA: *P*^d^		
		4 weeks	8 weeks	12 weeks	Group	Time	Interaction
Immune function parameters							
Con A-induced proliferation (stimulation index)^b, c^	Control	− 24.6 ± 37.7	− 1.3 ± 62.2	− 26.9 ± 40.7	0.04	0.10	0.34
	HK L-137	− 6.3 ± 50.3*	− 1.4 ± 47.4	− 5.7 ± 75.9^†^			
Th1:Th2 ratio	Control	3.0 ± 36.9	− 16.8 ± 22.9	23.6 ± 36.6	0.53	< 0.01	0.70
	HK L-137	− 3.5 ± 28.8	− 18.5 ± 29.3	24.7 ± 30.5			

*Con A* concanavalin A, *Th1* type 1 T helper, *Th2* type 2 helper

^a^Mean ± SD of % changes in immune parameters at each time point vs. baseline

^b^Ratio of Con A-induced proliferation to unstimulated proliferation

^c^Stimulation index (control group: *n* = 48, 47, 49 in the 4, 8, 12 weeks)

^d^Significant differences were evaluated by two-way ANOVA

*P < 0.05, ^†^*P* < 0.10: significant difference of the mean value vs. the control group (unpaired Student’s *t* test or Welch’s *t* test)

### Effects of HK L-137 on serum inflammatory and lipid markers

There was no significant difference of BMI between the control group and the HK L-137 group during the study period (data not shown). In the HK L-137 group, the leucocyte count of 1 subject at 12 weeks was unavailable for analysis because of coagulation. In addition, serum CRP levels ≥ 1 mg/dL (2 at 4 weeks and 1 at 12 weeks in the control group; 2 at 4 weeks, 4 at 8 weeks, 3 at 12 weeks in the HK L-137 group) were excluded from analysis because of reflecting acute inflammation. The decreases in the serum levels of aspartate transaminase (AST) and alanine aminotransferase (ALT), biomarkers of hepatic inflammation, were significantly larger in the HK L-137 group than in the control group during the study period (*P* = 0.02 and *P* = 0.02, respectively), and the decrease of the leukocyte count also tended to be larger in the HK L-137 group (*P* = 0.08) (Table 3). In addition, the decrease of AST (at 4 and 12 weeks), ALT (at 4 weeks), and the leukocyte count (at 4 weeks) were significantly larger in the HK L-137 group compared with the control group (Table 3). Among the serum lipid markers, TC and LDL-C increased in the control group during the study due to seasonal variation [32, 33], while these increases were somewhat suppressed in the HK L-137 group, leading to significant differences in the changes of TC and LDL-C between the two groups at 12 weeks (Table 3). There were no significant differences in the changes of other parameters between the two groups.

**Table 3:** Changes of various parameters from baseline

		Change from baseline^a^			Repeated two-way ANOVA: P^d^		
		4 weeks	8 weeks	12 weeks	Group	Time	Interaction
Blood parameters							
AST (units/L)	Control	3.1 ± 10.6	0.6 ± 4.0	1.1 ± 4.3	0.02	0.54	0.12
	HK L-137	− 1.3 ± 4.9*	0.0 ± 10.9	− 1.2 ± 5.1*			
ALT (units/L)	Control	5.2 ± 16.0	2.2 ± 9.9	3.3 ± 8.9	0.02	0.47	0.43
	HK L-137	− 0.1 ± 8.1*	0.1 ± 8.3	− 0.1 ± 8.1^†^			
CRP (mg/dL)^b^	Control	0.014 ± 0.090	− 0.005 ± 0.079	− 0.004 ± 0.098	0.79	0.03	0.19
	HK L-137	− 0.007 ± 0.176	0.010 ± 0.139	− 0.030 ± 0.174			
Glucose (mg/dL)	Control	− 1.84 ± 7.65	1.20 ± 5.54	0.16 ± 6.35	0.47	< 0.01	0.66
	HK L-137	− 0.85 ± 6.81	2.40 ± 5.77	0.13 ± 5.97			
TC (mg/dL)	Control	3.3 ± 23.5	0.4 ± 20.4	7.7 ± 17.4	0.49	0.01	< 0.05
	HK L-137	6.9 ± 21.1	− 2.6 ± 21.9	0.2 ± 19.0*			
HDL-C (mg/dL)	Control	0.8 ± 6.2	0.7 ± 6.0	2.3 ± 5.9	0.75	< 0.10	0.94
	HK L-137	1.4 ± 7.2	1.0 ± 6.5	2.3 ± 5.8			
LDL-C (mg/dL)	Control	6.5 ± 22.5	5.2 ± 17.7	9.1 ± 17.4	0.38	0.13	0.06
	HK L-137	8.4 ± 19.9	2.0 ± 21.9	1.8 ± 17.0*			
Leukocyte count (/mL)^c^	Control	0.16 ± 1.31	0.02 ± 1.11	0.10 ± 1.12	0.08	0.91	0.29
	HK L-137	− 0.45 ± 1.51*	− 0.29 ± 1.91	− 0.44 ± 1.77^†^			
TNF-α (pg/mL)	Control	− 0.30 ± 1.34	− 0.02 ± 0.81	− 0.05 ± 0.79	0.69	< 0.01	0.21
	HK L-137	− 0.52 ± 0.83	0.05 ± 0.92	− 0.10 ± 0.87			
TG (mg/dL)	Control	− 5.4 ± 51.5	3.1 ± 35.1	3.2 ± 52.7	0.95	0.49	0.78
	HK L-137	− 0.9 ± 64.0	− 0.7 ± 74.4	4.5 ± 88.8			

*AST* aspartate transaminase, *ALT* alanine transaminase, *CRP* C-reactive protein, *TC* total cholesterol, *HDL-C* high-density lipoprotein cholesterol, *LDL-C* low-density lipoprotein cholesterol, *TNF* tumor necrosis factor, *TG* triglyceride

^a^Mean ± SD of the change from baseline at each time point

^b^CRP: *t* test (control group: *n* = 47, 49, 48; HK L-137 group: *n* = 45, 43, 44 at 4, 8, 12 weeks, respectively), repeated two-way ANOVA (control group: *n* = 46; HK L-137 group: *n* = 42)

^c^Leukocyte count: *t* test (HK L-137 group: *n* = 46 at 12 weeks), repeated two-way ANOVA (control group: *n* = 49; HK L-137 group: *n* = 46)

^d^Significant differences were evaluated by repeated two-way ANOVA

*P < 0.05, ^†^*P* < 0.10: significant difference of the mean value vs. the control group (unpaired Student’s *t* test or Welch’s *t* test)

### Analysis stratified by serum CRP

To investigate the possible mechanism underlying the effect of HK L-137 on liver inflammation and cholesterol metabolism, we assessed the influence of inflammation by CRP-stratified analysis. In either low or high CRP subjects, BMI showed no significant difference between the control and HK L-137 groups during the study period (data not shown). Among the high CRP subjects, the decreases of AST, ALT, and the leukocyte count were significantly larger (*P* < 0.01, *P* = 0.01, and *P* = 0.02) in the HK L-137 group compared with the control group during the study period (Table 4). In addition, the decreases of AST at 4, 8 and 12 weeks, ALT at 4 and 12 weeks, and the leukocyte count at 4 and 12 weeks were significantly larger in the HK L-137 group than in the control group (Table 4). Seasonal increases in the levels of TC and LDL-C were observed in the control group, but not in the HK L-137 group, resulting in significant differences between the two groups at 12 weeks (Table 4). In contrast, among the low CRP subjects, there were no significant differences in the changes of AST, ALT, TC, and LDL-C between the HK L-137 group and the control group at any time point or across the entire study (Table 4).

**Table 4:** Analysis of lipid parameters and inflammatory markers stratified by CRP

		Change from baseline^a^			Repeated two-way ANOVA: P^d^		
		4 weeks	8 weeks	12 weeks	Group	Time	Interaction
CRP ≥ 0.045 mg/dL^c^							
AST (unit/L)	Control	2.7 ± 5.8	0.9 ± 4.5	2.2 ± 4.1	< 0.01	0.21	0.55
	HK L-137	− 2.3 ± 5.3*	− 2.7 ± 4.9*	− 2.2 ± 5.2*			
ALT (unit/L)	Control	5.6 ± 14.8	2.9 ± 9.5	4.7 ± 9.6	0.01	0.61	0.61
	HK L-137	− 1.8 ± 7.1*	− 1.6 ± 7.5^†^	− 1.1 ± 8.9*			
TC (mg/dL)	Control	5.0 ± 22.3	− 0.8 ± 18.4	8.4 ± 13.5	0.19	0.06	0.37
	HK L-137	2.4 ± 18.4	− 5.3 ± 23.7	− 2.6 ± 19.8*			
HDL-C (mg/dL)	Control	1.4 ± 5.9	0.4 ± 5.6	2.1 ± 5.5	0.87	0.21	0.70
	HK L-137	0.7 ± 5.4	1.2 ± 5.1	2.6 ± 5.0			
LDL-C (mg/dL)	Control	6.6 ± 20.5	3.9 ± 15.1	9.6 ± 13.8	0.21	0.32	0.33
	HK L-137	4.7 ± 17.0	− 1.0 ± 25.5	− 0.8 ± 19.2*			
Leukocyte count (/mL)	Control	0.50 ± 0.98	0.54 ± 0.85	0.40 ± 0.81	0.02	0.60	0.98
	HK L-137	− 0.50 ± 1.74*	− 0.43 ± 2.27^†^	− 0.63 ± 1.94*			
CRP < 0.045 mg/dL^b^							
AST (units/L)	Control	3.4 ± 13.4	0.3 ± 3.5	0.2 ± 4.3	0.94	0.51	0.19
	HK L-137	0.1 ± 4.0	3.5 ± 15.2	− 0.1 ± 4.9			
ALT (units/L)	Control	5.0 ± 17.2	1.7 ± 10.4	2.2 ± 8.3	0.68	0.56	0.72
	HK L-137	2.5 ± 8.8	2.3 ± 9.3	1.3 ± 7.3			
TC (mg/dL)	Control	1.9 ± 24.8	1.4 ± 22.1	7.2 ± 20.2	0.76	0.24	0.14
	HK L-137	11.2 ± 23.8	0.7 ± 20.3	3.5 ± 18.7			
HDL-C (mg/dL)	Control	0.3 ± 6.5	0.9 ± 6.4	2.4 ± 6.4	0.70	0.45	0.67
	HK L-137	2.2 ± 9.4	1.1 ± 8.2	2.4 ± 6.9			
LDL-C (mg/dL)	Control	6.5 ± 24.4	6.3 ± 19.8	8.7 ± 20.0	0.91	0.43	0.20
	HK L-137	12.5 ± 23.0	5.9 ± 17.4	5.0 ± 14.6			
Leukocyte count (/mL)	Control	− 0.13 ± 1.48	− 0.40 ± 1.13	− 0.14 ± 1.28	0.19	0.60	0.07
	HK L-137	− 0.07 ± 0.66	0.27 ± 0.73*	0.21 ± 0.88			

*AST* aspartate transaminase, *ALT* alanine transaminase, *CRP* C-reactive protein, *HDL-C* high-density lipoprotein cholesterol, *TC* total cholesterol, *LDL-C* low-density lipoprotein cholesterol

^a^Mean ± SD of the change from baseline at each time point

^b^Control group: *n* = 27; HK L-137 group: *n* = 20, except for leukocyte count (control: *n* = 27; HK L-137: *n* = 19)

^c^Control group: *n* = 22; HK L-137 group: *n* = 25

^d^Significant differences were evaluated by repeated two-way ANOVA

*P < 0.05, ^†^*P* < 0.10: significant difference of the mean value vs. the control group (unpaired Student’s *t* test or Welch’s *t* test)

### Safety

A total of 38 adverse events were recorded during the study. Among 17 adverse events in the control group, there were four of influenza, two of inflammatory symptoms, two of pain, one of hyperesthesia, two of high ALT levels, one of a high AST level, two of high gamma-glutamyl transpeptidase levels, one of a high alkaline phosphatase level, and two of high creatine kinase levels. Among 21 adverse events in the HK L-137 group, there were four of influenza, four of common cold symptoms, two of inflammatory symptoms, two of digestive symptoms, one of pruritus, one of palpitations, three of high creatine kinase levels, one of a high uric acid level, one of a high insulin level, one of high urinary protein, and one of hypertension. All adverse events were mild, and were judged to be unrelated to the study treatment. Among the safety parameters, the pulse rate, serum calcium level, and platelet count were significantly higher in the HK L-137 group than in the control group, while total bilirubin and blood urea nitrogen were significantly lower, but all values were within the corresponding reference ranges. Among male subjects, the red blood cell count, hemoglobin, and hematocrit were significantly higher in the HK L-137 group than in the control group, but all values were within the reference ranges.

## Discussion

In the present study, oral intake of HK L-137 significantly augmented the proliferative response of PBMCs to Con A in overweight healthy subjects, which was a similar finding to our previous reports. Among the biomarkers of inflammation, the decreases of AST and ALT were significantly larger and that of the leukocyte count also tended to be larger in the HK L-137 group than in the control group throughout the study. In addition, biomarkers of lipid metabolism such as TC and LDL-C showed a decrease in the HK L-137 group compared with the control group at 12 weeks. These findings suggested that daily intake of HK L-137 improved hepatic inflammation and serum cholesterol in overweight subjects.

The intestinal barrier normally prevents translocation of lipopolysaccharide (LPS) derived from microbiota or the diet, so deterioration of this barrier results in a chronic increase of plasma LPS levels in obese animal or humans [34–37]. After translocation, LPS binds to lipoproteins such as chylomicrons, HDL, LDL, and VLDL. The complexes thus formed are preferentially transported to the liver and incorporated by Kupffer cells, leading to increased production of TNF-α [38–40]. Recently, there have been a number of reports that lactic acid bacteria improve the gut barrier in obesity. *L. gasseri* SBT2055 was found to improve the integrity of the intestinal barrier in obese mice, reducing entry of LPS from the intestine, and also decreased body weight, visceral fat mass, and inflammation [41]. Hsieh et al. demonstrated that both viable and heat-killed *L. reuteri* GMNL-263 reversed impairment of the intestinal barrier in obese rats [42]. In the present study, intake of HK L-137 improved hepatic and systemic inflammation, especially in the subjects with higher CRP levels who potentially have impairment of the intestinal barrier as a cause of inflammation (Table 4). Since HK L-137 has been shown to improve the morphology of intestinal villi and epithelial cells in broiler chickens [43], it is possible that it restored the intestinal barrier in our subjects, leading to improvement of systemic inflammation (leukocyte count) and hepatic inflammation (AST and ALT). The novel finding of this study was that intake of HK L-137 decreased biomarkers of hepatic inflammation. To our knowledge, this is the first study to show that intake of lactobacilli can reduce AST and ALT levels in healthy human subjects. It has been reported that slight elevation of serum ALT, even within the normal range, can predict a higher risk of type 2 diabetes, metabolic syndrome, and coronary heart disease [44–47]. While the reduction of AST and ALT by HK L-137 was within the normal range in this study, these reports suggest that lowering serum ALT within the normal range may reduce the risk of type 2 diabetes, metabolic syndrome, and coronary heart disease. Because we did not measure LPS, further investigation is needed to clarify the anti-inflammatory mechanism of HK L-137.

It was reported that a high-fat diet weakens the intestinal barrier in mice by reducing the expression of epithelial tight junction proteins such as ZO-1 [35]. Cell wall fractions of *Enterococcus hirae* (e.g., lipoteichoic acid) have been demonstrated to promote recovery of ZO-1 protein expression and transepithelial resistance in TNF-alpha-treated Caco-2 cells [48]. Miyauchi et al. also showed that both viable and heat-killed *L. rhamnosus* OLL2838 protected mice from dextran sulfate sodium (DSS)-induced colitis, along with elevation of intestinal ZO-1 gene expression that had been decreased by DSS treatment [49]. HK L-137 was shown to have a beneficial effect on DSS-induced colitis in mice [50], suggesting that it may improve the intestinal barrier by normalizing ZO-1 expression.

HK L-137 improved TC and LDL-C levels in the present study, especially in the subjects with high serum CRP, an indicator of total inflammation induced by endogenous and exogenous stimuli (Table 4). It was reported that LPS increases serum cholesterol in Syrian hamsters by inducing hepatic cholesterol synthesis [51], while the TC level showed a significant correlation with the endotoxin level in healthy subjects [52]. Thus, HK L-137 might decrease TC and LDL-C levels by improving the intestinal epithelial barrier function and inhibiting the translocation of endotoxin.

In conclusion, we found that daily intake of HK L-137 enhanced T-cell responses and suppressed hepatic inflammation and serum cholesterol in overweight subjects. It is possible that HK L-137 may be useful for prevention/treatment of metabolic dysfunction in persons at risk of inflammation.

## Abbreviations

ALT: Alanine aminotransferase
ANOVA: Analysis of variance
AST: Aspartate transaminase
BMI: Body mass index
CD: Cluster of differentiation
Con A: Concanavalin A
CRP: C-reactive protein
DSS: Dextran sulfate sodium
HDL-C: High-density lipoprotein cholesterol
HK L-137: Heat-killed *Lactobacillus plantarum* L-137
IFN: Interferon
IL: Interleukin
LDL-C: Low-density lipoprotein cholesterol
LPS: Lipopolysaccharide
PBMCs: Peripheral blood mononuclear cells
TC: Total cholesterol
TG: Triglyceride
Th: T helper
TNF: Tumor necrosis factor
VLDL: Very-low-density lipoprotein
